# Radiation therapy induces immunosenescence mediated by p90RSK

**DOI:** 10.3389/fcvm.2022.988713

**Published:** 2022-11-07

**Authors:** Masaki Imanishi, Haizi Cheng, Sivareddy Kotla, Anita Deswal, Nhat-Tu Le, Eduardo Chini, Kyung Ae Ko, Venkata S. K. Samanthapudi, Ling-Ling Lee, Joerg Herrmann, Xiaolei Xu, Cielito Reyes-Gibby, Sai-Ching J. Yeung, Keri L. Schadler, Syed Wamique Yusuf, Zhongxing Liao, Roza Nurieva, El-ad David Amir, Jared K. Burks, Nicolas L. Palaskas, John P. Cooke, Steven H. Lin, Michihiro Kobayashi, Momoko Yoshimoto, Jun-ichi Abe

**Affiliations:** ^1^Department of Cardiology, The University of Texas MD Anderson Cancer Center, Houston, TX, United States; ^2^Center for Stem Cell and Regenerative Medicine, Institute of Molecular Medicine, McGovern Medical School, University of Texas Health Science Center at Houston, Houston, TX, United States; ^3^Department of Cardiovascular Sciences, Houston Methodist Research Institute, Houston, TX, United States; ^4^Department of Anesthesiology and Perioperative Medicine, Mayo Clinic, Jacksonville, FL, United States; ^5^Division of Preventive Cardiology, Cardio Oncology Clinic, Department of Cardiovascular Medicine, Mayo Clinic, Rochester, MN, United States; ^6^Department of Biochemistry and Molecular Biology, Mayo Clinic, Rochester, MN, United States; ^7^Department of Emergency Medicine, The University of Texas MD Anderson Cancer Center, Houston, TX, United States; ^8^Department of Pediatric Research, The University of Texas MD Anderson Cancer Center, Houston, TX, United States; ^9^Department of Radiation Oncology, The University of Texas MD Anderson Cancer Center, Houston, TX, United States; ^10^Division of Basic Science, Department of Immunology, The University of Texas MD Anderson Cancer Center, Houston, TX, United States; ^11^Astrolabe Diagnostics, Inc., Fort Lee, NJ, United States; ^12^Division of Center Medicine, Department of Leukemia, The University of Texas MD Anderson Cancer Center, Houston, TX, United States

**Keywords:** radiotherapy, immunosenescence, p90RSK, CD38, T-bet

## Abstract

Radiation therapy (RT) to the chest increases the patients’ risk of cardiovascular disease (CVD). A complete understanding of the mechanisms by which RT induces CVD could lead to specific preventive, therapeutic approaches. It is becoming evident that both genotoxic chemotherapy agents and radiation induce mitochondrial dysfunction and cellular senescence. Notably, one of the common phenotypes observed in cancer survivors is accelerated senescence, and immunosenescence is closely related to both cancer risk and CVD development. Therefore, suppression of immunosenescence can be an ideal target to prevent cancer treatment-induced CVD. However, the mechanism(s) by which cancer treatments induce immunosenescence are incompletely characterized. We isolated peripheral blood mononuclear cells (PBMCs) before and 3 months after RT from 16 thoracic cancer patients. We characterized human immune cell lineages and markers of senescence, DNA damage response (DDR), efferocytosis, and determinants of clonal hematopoiesis of indeterminant potential (CHIP), using mass cytometry (CyTOF). We found that the frequency of the B cell subtype was decreased after RT. Unsupervised clustering of the CyTOF data identified 138 functional subsets of PBMCs. Compared with baseline, RT increased TBX21 (T-bet) expression in the largest B cell subset of Ki67^–^/DNMT3a^+^naïve B cells, and T-bet expression was correlated with phosphorylation of p90RSK expression. CD38 expression was also increased in naïve B cells (CD27^–^) and CD8^+^ effector memory CD45RA T cells (T_EMRA_). *In vitro*, we found the critical role of p90RSK activation in upregulating (1) CD38^+^/T-bet^+^ memory and naïve B, and myeloid cells, (2) senescence-associated β-gal staining, and (3) mitochondrial reactive oxygen species (ROS) after ionizing radiation (IR). These data suggest the crucial role of p90RSK activation in immunosenescence. The critical role of p90RSK activation in immune cells and T-bet induction in upregulating atherosclerosis formation has been reported. Furthermore, T-bet directly binds to the CD38 promoter region and upregulates CD38 expression. Since both T-bet and CD38 play a significant role in the process of immunosenescence, our data provide a cellular and molecular mechanism that links RT-induced p90RSK activation and the immunosenescence with T-bet and CD38 induction observed in thoracic cancer patients treated by RT and suggests that targeting the p90RSK/T-bet/CD38 pathway could play a role in preventing the radiation-associated CVD and improving cancer prognosis by inhibiting immunosenescence.

## Introduction

Radiation therapy (RT) is an important component of managing primary thoracic malignancies, e.g., lung cancer, breast cancer and lymphoma. The delayed effects of RT on the cardiovascular system causes substantial morbidity and mortality in cancer survivors (1). Cardiovascular disease (CVD) is a leading cause of premature morbidity and mortality among breast cancer (2–4), Hodgkin or non-Hodgkin lymphoma (5–8), and lung cancer (9, 10) survivors more than 5 years after their diagnosis and treatment. RT to the chest in Hodgkin lymphoma increases the patients’ risk of CVD by about sevenfold, based on the results of multivariate analyses controlling for other risk factors (11). The incidence of major coronary events increases linearly by 7.4% per Gy in breast cancer survivors (1). Modern RT techniques significantly decrease the doses of radiation to the heart, but in breast cancer patients, the heart may still receive doses of 1–5 Gray (Gy) (1). A more complete understanding of the mechanisms by which RT induces CVD could lead to specific preventive therapeutic approaches.

Risk factors for CVD are generally greater for cancer survivors than the general population, with higher values for mean body mass index, C-reactive protein and low density lipoprotein cholesterol (12). One of the common phenotypes observed in cancer survivors is accelerated senescence (12–14). It is becoming evident that genotoxic chemotherapy agents and radiation induce mitochondrial dysfunction and cellular senescence, i.e., stress-induced premature senescence (SIPS). DNA damaging agents can cause SIPS in a relatively short period (usually 3–10 days) with or without significant telomere shortening (15). Most DNA damage is repaired by DNA damage response (DDR) mechanisms within 24 h after stress (16), but, in contrast, telomeric DNA damage persists for months (17). Therefore, telomeric DNA damage-induced SIPS may be a feasible explanation for the late or delayed CVD effects triggered by cancer treatments.

Although cellular senescence can lead to cell cycle arrest, senescent cells remain metabolically active and secrete a variety of factors, including inflammatory cytokines, chemokines, growth factors, angiogenic factors, nitric oxide (NO), prostaglandin E2 (PGE2), and reactive oxygen species (ROS), constituting a senescence-associated secretory phenotype (SASP). To maintain tissue homeostasis, removing senescent cells promptly is crucial. The SASP cytokines can recruit immune cells to clear the senescent cells by efferocytosis. On the other hand, “immunosenescence” refers to the decline in the immune response with aging (18), including impaired clearance of senescent cells. The critical role of immunosenescence in regulating both tumorigenesis (19) and CVD (20) has been suggested, but the exact regulatory mechanism remains unclear.

As cells age or are exposed to mutagens, they may acquire mutations that lead to clonal expansion without malignant transformation. In the hematopoietic system, this process is known as clonal hematopoiesis of indeterminant potential (CHIP). In over 70% of people with CHIP, somatic mutations of *TET2*, *DNMT3A*, *ASXL1*, and *JAK2* are commonly observed, and all but one (*JAK2*) are loss of function mutations (21, 22). CHIP is associated with a more than twofold increase in risk of CVD (22, 23), which is likely mediated through inflammatory pathways (22, 24). Among survivors of solid malignancies, CHIP-associated mutations are common and are detectable in around 30% of individuals in their 7th decade of age, and may adversely impact overall survival (25). Oncologic therapies, such as RT, are frequently dose-limited based on toxicity to normal organs—organs that may already have impaired function in the presence of CHIP-associated mutations (22, 26–28). It is undefined whether the presence of CHIP in patients undergoing RT for thoracic malignancies contributes to an increased risk of radiotherapy-induced CVD.

The critical role of NAD^+^ depletion during aging in regulating metabolic dysfunction is well established (29). Previously, we reported that NAD^+^ was depleted after IR *via* PARP activation, triggering persistent PISP in myeloid cells (30). In addition to PARP activation, CD38 may be involved in NAD^+^ depletion during aging (31). CD38 is a NAD glycohydrolase, and the levels and activity of CD38 were significantly increased by aging, leading to NAD^+^ depletion (31). CD38 is expressed mainly in immune cells, and recently, Chini et al. have reported that senescence-induced inflammation promoted CD38 accumulation and caused NAD^+^ depletion (32). Taken together, these reports suggested that both PARP activation and CD38 expression promote NAD^+^ depletion during the aging process.

T-bet (official gene name: *TBX21*) is an immune cell-specific member of the T-box family of transcription factors. In B cells, antigen stimulation activates B cell proliferation and induces affinity maturation and class switch recombination to produce high-affinity antibodies, which show different immune effector functions (33). T-bet induces Iγ2A transcripts and promotes IFNγ-mediated class-switching to the IgG2a isotype, which plays a critical role in the pathogenesis of humoral autoimmunity and protection against pathogens (34). Furthermore, T-bet promotes the survival of antigen-specific IgG2a^+^/CD38^hi^ memory B cells by upregulating mature B cell receptor transcription (35). Also, T-bet drives the migration of memory IgG2a^+^ B cell to sites of inflammation by increasing CXCR3 expression (36). Importantly, the age-associated B cell (ABC) is characterized by T-bet expression and increases overall inflammatory state (37). Although systemic T-bet depletion inhibited atherosclerosis formation (38), the role of T-bet in B cells on atherosclerosis formation remains unclear.

Previously, we reported the role of p90RSK activation in SASP after RT. IR increases p90RSK activation in myeloid cells and forms a nucleus-mitochondria positive feedback loop with p90RSK-mediated ERK5 S496 phosphorylation, leading to persistent mitochondrial (mt) ROS production and subsequent telomere DNA damage. We found that poly (ADP-ribose) polymerase activation induced by telomere DNA damage promoted nicotinamide mononucleotide (NAD^+^) depletion and consequent mitochondrial dysfunction, which elicited mtROS production, activated redox-sensitive kinase of p90RSK, and formed a positive feedback loop. Therefore, p90RSK activation played a crucial role in inducing SASP.

Although RT may induce immunosenescence (39), the mechanisms remain incompletely defined. Therefore, in this study, we use multiparameter mass cytometry (CyTOF) to explore the association of RT with various senescence-related factors and determinants of senescence including DDR -related markers, efferocytosis, and CHIP-associated drivers in PBMCs from thoracic cancer patients before and 3 months after RT.

## Materials and methods

### Patients selection

The study was approved by the Institutional Review Board of the University of Texas MD Anderson Cancer Center (#PA16-0971). The inclusion criteria of this study were as follows: (1) ≥ 18 years of age, (2) *Karnofsky Performance Scale* ≥ 70, (3) To receive radiation therapy (RT) with computed tomography (CT)-based treatment planning that will involve delivery of dose to the heart, (3) An estimated mean heart dose of at least 6 Gy and mean left ventricular dose of at least 5 Gy, as estimated by the treating radiation oncologist at the time of simulation.

The exclusion criteria of this study were as follows: (1) Unable or unwilling to give written informed consent, (2) Previous history of RT to the thorax or breast, (3) Allergy to gadolinium, (4) Implanted device that is non-MRI compatible, including cardiac devices and neurostimulators, (5) Pregnant or breast-feeding, (6) Atrial fibrillation or frequent ventricular or atrial premature beats, (7) GFR < 60 mL/min according to the Modification of Diet in Renal Disease equation, (8) Personal history of coronary artery disease or myocardial disease, (9) Personal history of hypertension, requiring > 1 antihypertensive agent to maintain blood pressure < 140/90, (10) Valvular stenosis or regurgitation of > moderate severity, (11) Heart failure (baseline NYHA > 2), (12) Systolic BP < 90 mmHg (15) Pulse < 50/min, (16) History of pulmonary hypertension or elevated right ventricular systolic pressures by echocardiogram, (17) Renal failure necessitating dialysis, (18) Suspicion or diagnosis of amyloidosis, (19) Suspicion or diagnosis of hemochromatosis, (20) Unable to obtain an MRI scan with gadolinium for any other reason that is not listed above.

### Panel design and metal conjugation of antibodies

We generated a CyTOF panel which includes antibodies against CHIP drivers, SASP-related proteins, and various cell surface markers for PBMCs such as the markers described in the previous report (40). We used 37 antibodies, Ir DNA-Intercalator (Cell-ID Intercalator-Ir; Fluidigm #201192A) for nucleus staining and Rh DNA-intercalator (Cell-ID Intercalator-103Rh; Fluidigm #201103A) for viability staining, as listed in Supplementary Table 1. We designed the panel using Maxpar Panel Designer software (Fluidigm). The antibodies listed in Supplementary Table 1, except for the metal-conjugated antibodies that were purchased from Fluidigm, BioLegend, BD Biosciences, and Miltenyi, were conjugated to lanthanides using the Maxpar X8 Multimetal Labeling Kit (Fluidigm) or cadmium using the Maxpar MCP9 Antibody Labeling Kit (Fluidigm) according to the manufacturer’s protocol. To determine optimal antibody concentrations, three different concentrations of each metal-labeled antibody were tested by CyTOF before being used in analyses.

The 90 kDa ribosomal S6 kinases (p90RSKs) are a group of serine/threonine kinases consisting of 4 RSK isoforms (RSK1-4). The expressions of p90RSK1-4 isoforms in human are ubiquitous, and all of them express in immune cells.^1^ The p-p90RSK antibody recognizes the phosphorylation of p90RSK1 S380 site, which has a homology between p90RSK1-3 at LFRGFSpFVA, and p90RSK4 at SpFVA. Therefore, at least this antibody can recognize p90RSK1-3 isoforms.

### Cytometry sample preparation and staining

Whole blood was collected at two different time points, before (baseline) and 3 months after RT, from patients (*n* = 16) with thoracic cancer treated at The University of Texas MD Anderson Cancer Center (Supplementary Table 2). PBMCs were isolated using Ficoll-Paque Plus (Fisher #45-001-750) according to the manufacturer’s protocol and were cryopreserved in liquid nitrogen until analysis. Cryopreserved PBMC samples were thawed in RPMI containing 5% pooled human serum (PHS), 10 mM HEPES, 0.2% gentamicin, and 2.5 μ/mL benzonase nuclease, washed once with the same medium and repelleted by centrifugation at 300 g at 4^°^C). One million cells were applied to the viability staining with Cell-ID Intercalator-103Rh and washed with Maxpar Cell staining buffer (Fluidigm #201068). For cell fixation, the cells were resuspended in 1x Fix I Buffer (Fluidigm #201065) and start the barcoding step using Cell-ID 20 Plex Pd Barcoding Kit (Fluidigm #201060). All barcoded samples were combined, and the staining procedure was performed according to protocol, i.e., Maxpar Phosphoprotein Staining with Fresh Fix (Fluidigm PN400278A4 protocol). The cells were blocked by Human TruStain FcX (BioLegend #422302), followed by cell surface staining for cell-type identification. After the wash with Maxpar Cell staining buffer, the cells were chilled on ice and incubated at 4*^o^*C with methanol for permeabilization, followed by two washes with Maxpar Cell staining buffer. The cells were incubated with metal-labeled antibodies against cytoplasmic proteins followed by two washes. For the final fixation, the cells were incubated with 1.6% formaldehyde, followed by the incubation with Cell-ID Intercalator-Ir for nucleus staining. After washing twice with Maxpar Cell Staining Buffer and once with Maxpar water (Fluidigm #201069), the cells were filtered to make a single cell suspension and centrifuged. After adding EQ beads (Fluidigm #201078), CyTOF data were acquired on a Helios mass cytometer (Fluidigm).

### Data acquisition for cytometry

CyTOF data were acquired on a Helios mass cytometer (Fluidigm) and were analyzed using the Astrolabe Cytometry Platform (Astrolabe Diagnostics, Inc.). Single-cell data have been clustered using the FlowSOM R package (41) and labeled using the Ek’Balam algorithm (42). Cell subset definitions were followed as previously described (43, 44). Cluster labeling, method implementation, and visualization were done through the Astrolabe Cytometry Platform (Astrolabe Diagnostics, Inc.).

### Irradiation of human peripheral blood mononuclear cells followed by flow cytometry

Human peripheral blood mononuclear cells (hPBMC) were seeded into a 48-well plate in culture medium (RPMI 1640 with 10%FBS, 10 mM HEPES, 1 × non-essential amino acid mix, 1 mM sodium pyruvate, 100 mg/mL streptomycin, 0.55 mM 2-mercaptoethanol) at a density of 2 million cells per well. Treatments were performed in triplicate for each group. We pre-treated the cells with FMK (RSK inhibitor Fmk, Soluble in DMSO, Axon Medchem Cat#1848) or DMSO for 1 h before irradiation. The cells were irradiated at 2 Gy while the non-irradiation control was set aside. Twenty-four hours after irradiation, we harvested the cells of each group and analyzed them on an LSRII instrument (Becton Dickinson). Flow CyTOF data were analyzed using FlowJo software. The following Cell surface markers were used: human CD3 (Biotin, clone: UCHT1, eBioscience), CD14 (Per-Cy5.5, clone: 63D3, BioLegend), CD19 (PE-Cy7, clone: HIB19, BioLegend), CD27 (PE, clone: O323, Tonbo), IgD (FITC, clone: MEM-59, BioLegend), CD38 (BUV395, clone: HB7, BD), and T-bet (Alexa Fluor 647, clone:4B10, Biolegend). Mouse IgG1 Isotype Control APC (clone:MOPC-21, Tonbo) was used as a negative control.

### Mitochondria-specific reactive oxygen species measurements

PBMC were washed with PBS and resuspended in RPMI 1640 supplemented with 10% FBS and 1% each of L-glutamine, sodium pyruvate, non-essential amino acids, 100 mg/ml streptomycin, 0.1 M HEPES, 55 μM β-mercaptoethanol. PBMC were seeded at a density of 2 × 10^5^/ml and cultured 6 h at 37^°^C and 5%CO2. After resting, cells were pretreated with and without FMK-MEA (10 μM)/1 h and later treated with IR (2Gy) for 24 h. After that, cell were washed with a medium two times and incubated with Mito sox (5 μM) (Molecular Probes, Invitrogen, Carlsbad, CA) for 20 min at 37°C. Then cells were incubated with anti-CD19 APC and anti-CD14 PerCp Cy5.5 at 4°C for 30 min. Autofluorescence of unstained cells was used as a control for each sample. Approximately 100,000 gated events were acquired for each sample on BD LSR II (Becton-Dickinson, San Jose, CA). Dead cells and debris were excluded based on forward scatter and side scatter measurements. The percentage of MitoSOX expression positive cells were quantified on CD19 APC -positive gated Cells or CD14 PerCp Cy5.5-positive gated cells in the PE channel analyzed using FlowJo software (TreeStar, Ashland, OR).

### Senescence associated β-galactosidase activity in cell cultures

After 24 h of ionizing radiation, PBMCs were incubated with 33 mM C12FDG (Cayman chemicals) for 30 min in a 37^°^C water bath. Cells were centrifuged at 500 g for 5 min, washed with Cell Staining buffer (BD Biosciences), and stained with anti-CD19-APC and anti-CD14-PerCPCy5.5 at 4^°^C for 30 min. Cells were then washed in Cell Staining buffer and resuspended in Cell Staining buffer and analyzed by a BD LSR II Flow cytometer. Gates were set as follows: Dead cells and debris were excluded based on forward scatter and side scatter measurements, B cells (CD19^+^), Monocytes (CD14^+^) and analyzed for C 12 FDG fluorescence. Data were analyzed with FlowJo software (v.10).

### Statistical analysis

The multidimensional scaling (MDS) map was generated using the cmdscale R package (45). Differential abundance analysis was done using the edgeR R package (46, 47) as previously described (48). Differential expression analysis was done using the limma R package (49), as described previously (50). The frequency data within –log(FDR) > 0.5 and log [fold change (FC)] < –1 or 1 < log(FC) were considered as significant. The expression data within adjusted *P*-value (adj. *P*-Val) < 0.05 were considered as significant. For the adj. *P*-Val, limma R package is using the Benjamini-Hochberg correction.

We determined differences between 2 independent groups using the Student’s *t*-test (2-tailed) and, when applicable, 1-way analysis of variance followed by Bonferroni *post-hoc* testing for inter- group comparisons using GraphPad Prism (GraphPad Software, San Diego, CA, USA). We used Welch’s analysis for evaluating the equal variance of each group. *P*-values < 0.05 were considered statistically significant.

## Results

### The responses of immune cells after radiation treatment in cancer patients

**FIGURE 1:**
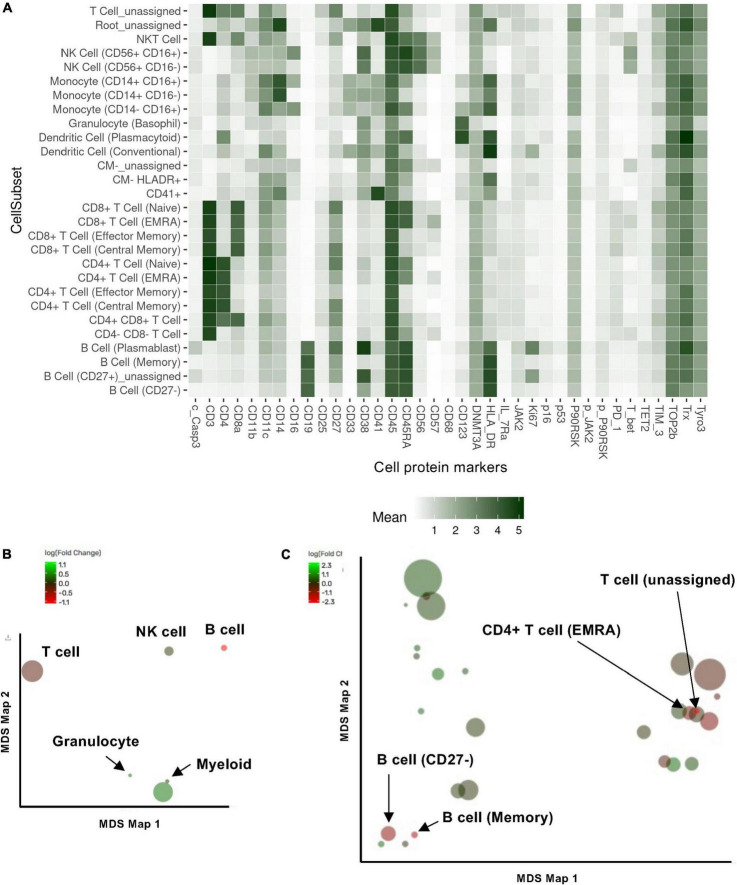
Cell subset using the Astrolabe platform. The FlowSOM algorithm clustered cells and labeled subsets according to the canonical gating hierarchy. **(A)** Cell subset heat map. The rows represent cell subsets, and the columns represent cell protein expression. Tile intensity showed the median value in the subset according to the color key. **(B,C)** Multidimensional scaling (MDS) map for each cell type **(B)** and cell subsets clustered by Astrolabe platform as shown in **(A)**. Each bubble represents a cell type **(B)** or subset **(C)**, and the size of that bubble is determined by the median frequency of cells contained in that bubble across all samples analyzed. Each bubble is colored based on the magnitude of the fold change post-radiation therapy (RT) over baseline.

Previous studies showed that ionizing radiation and various cancer treatments induced a pro-inflammatory senescent phenotype in myeloid cells (30). To characterize how RT can change immune cell phenotypes in cancer patients using multiparameter mass CyTOF, we enrolled 16 patients with esophagus or lung cancer who were about to start RT (Supplementary Table 2). Blood was collected at the time of enrollment and 3 months after the initial radiation treatment, and peripheral blood mononuclear cells (PBMCs) were collected. We then evaluated the phenotypic changes of immune cells by multiparameter mass CyTOF with general cell surface markers and various senescent markers as described in the methods. Analysis using the Astrolabe platform (40) automatically labeled canonical immune cell subsets (Figure 1A and Supplementary Table 2). Astrolabe identified eleven T cell subsets [including CD4^+^ and CD8^+^ T cells, naïve T_EMRA_ (effector memory cells re-expressing CD45RA), effector memory, and central memory cells], three B cell subsets, five myeloid cell subsets, two NK cell subsets, and granulocytes. We found that only B cells significantly decreased after RT (Figure 1B). However, when we looked at each immune cell subset, we found four immune cell subsets: CD4^+^ T_EMRA_ cell, B cell (Memory), T cell (unassigned), and naïve B cell (CD27^–^) were significantly decreased after RT (Figure 1C). Our data suggest that RT may play a significant role in regulating B cell functions in both memory and naïve B cells, including antigen producing and presentation. Furthermore, CD4^+^ T_EMRA_ cells can show a cytotoxic phenotype with upregulation of CX3CR1 expression (51–54), which is related to CD4 and CD8 T cell cytotoxic potentials. Therefore, these data suggest the potential role of RT in regulating cytotoxic abilities of T cells.

### Additional subsets revealed using functional antibodies

**FIGURE 2:**
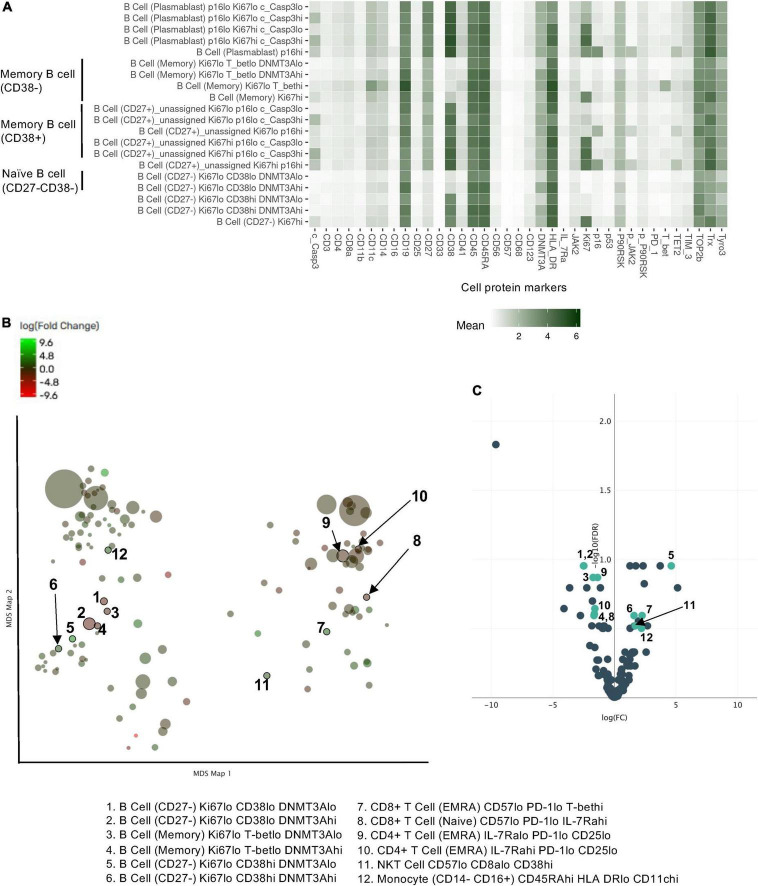
Profiling subsets using the Astrolabe platform. **(A)** Profiling subset heat map for B cells only. Refer to Supplementary Figure 1 for the complete heat map. Rows are cell subsets; columns are channels. Tile intensity is the median value measured in each channel in each profiling. **(B)** Profiling of every cell cluster (138 clusters) was visualized using MDS. Each cluster is colored based on the logarithm of the ratio of intensity in the post-RT sample to pre-RT (baseline) sample. **(C)** A volcano plot of identified cell clusters profiled in **(B)**, showing those with –log10 [the false discovery rate (FDR)] > 0.5, log(FC) < –1 or 1 < log(FC) and p < 0.05 (student’s *t*-test) after RT (pre-RT vs. post-RT as described in Figure 4A and Supplementary Figure 2) in light green. Once the frequency data within –log(FDR) > 0.5 and log[fold change (FC)] < –1 or 1 < log(FC) were considered as significant, 31 profiling subsets were changed by RT. Then 12 profiling subsets were picked out from the 31 subsets by student’s *t*-test.

**FIGURE 3:**
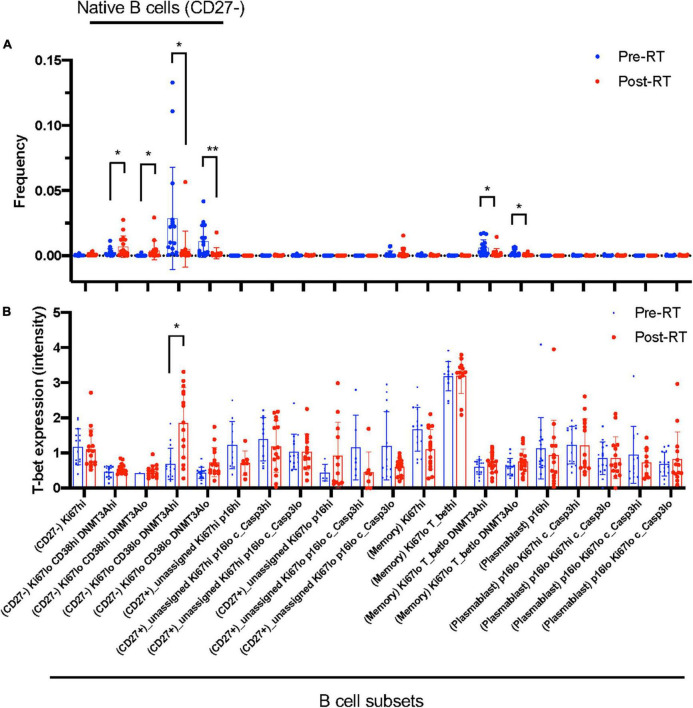
B cell profiling subset and T-bet expression. **(A)** The frequency of each B cell profiling subset for all the cells in an experiment at pre-RT and post-RT. *p < 0.05 (or **p < 0.01) by Student’s *t*-test, –log(FDR) > 0.5 and log(FC) < –1 or 1 < log(FC). **(B)** T-bet expression in each B cell profiling subset. The significant increase of T-bet expression after RT was observed only in the Ki67^–^DNMT3a^+^ naïve B cell subset. Mean ± SD, *adj. *P*-Val < 0.05.

We further included the staining data with functional antibodies, and the Astrolabe Platform derived 138 functional profiling subsets with unsupervised clustering by FlowSOM algorithm, which were labeled by the markers that provided the most significant separation among them (Figure 2 and Supplementary Figure 1). We found that the frequencies of 5 functional profiling subsets were significantly upregulated, and seven functional profiling subsets were downregulated after RT (Figures 2B,C, 3A and Supplementary Figure 2). Among 12 functional profiling subsets, we found that only three subsets, CD27^–^/Ki67^lo^/CD38^lo^/DNMT3a^hi^ B cells, CD4^+^/CD25^lo^/IL-7Ra^lo^/TET2^lo^ T_Naive_ cells and CD4^+^/Il-7Ra^lo^/PD-1^lo^/CD25^lo^ T_EMRA_ cells, showed > 1% frequency among total cells and were decreased after RT. These data suggest that the effects of RT on immune cells were relatively specific to a few unique functional subsets of naïve B cell and CD4^+^ T cell at 3 months after RT.

**FIGURE 4:**
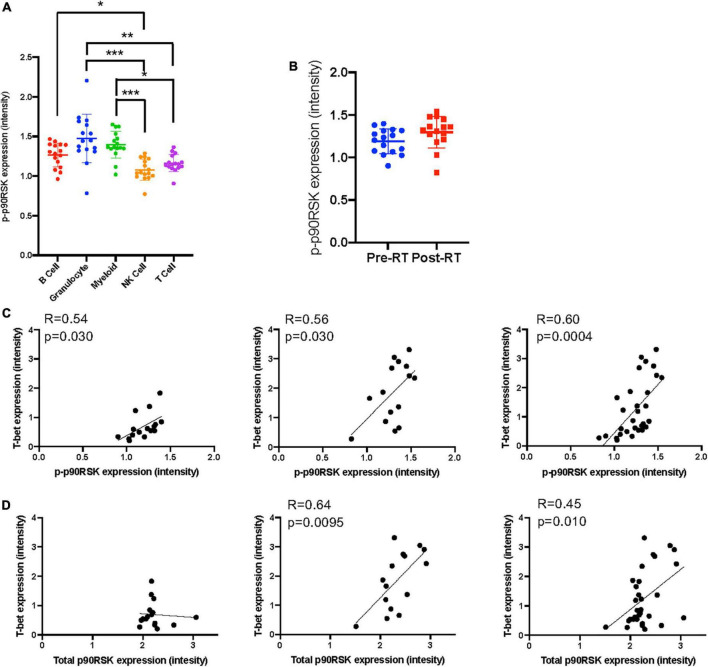
The relationship between T-bet and p90RSK in Ki67^–^DNMT3a^+^ naïve B cell subset. **(A)** p-p90RSK expression in each cell types. Mean ± SD, *p < 0.05, **p < 0.01, ***p < 0.001. Statistical significance was assessed by Dunn’s multiple comparisons test. **(B)** p-p90RSK expression in Ki67^–^DNMT3a^+^ naïve B cell subset showed no difference between pre-RT and post-RT. **(C)** The relationship between p-p90RSK and T-bet expression in Ki67^–^DNMT3a^+^ naïve B cell subset at pre-RT and post-RT. **(D)** The relationship between total p90RSK and T-bet expression in Ki67^–^DNMT3a^+^ naïve B cell subset at pre-RT and post-RT. Statistical significance and correlation were assessed by Pearson correlation.

### Radiation therapy uniquely induced T-bet and CD38 expression in Ki67^–^DNMT3a^+^ naïve B cell subset in cancer patients

Astrolabe revealed five functional subsets of B cells (CD27^–^). Among all the B cell subsets, CD27^–^/Ki67^lo^/CD38^lo^/DNMT3a^hi^ (Ki67^–^DNMT3a^+^ naïve B cell) was the largest, and CD27^–^/Ki67^lo^/CD38^lo^/DNMT3a^lo^ was the second-largest subset (Figure 3A). Although B cells did not highly express T-bet compared to other cell types in the baseline (Supplementary Figure 3) and the T-bet expression in Ki67^–^DNMT3a^+^ naïve B cell subset was not exceptionally high compared to different B cell subsets in the pre-RT (baseline) samples (Figure 3B and Table 1), we found that a significant increase of T-bet expression after RT was observed only in Ki67^–^DNMT3a^+^ naïve B cell subset (Figure 3B). Since it has been reported that most of the age- ABCs express T-bet (55), and we previously reported the role of p90RSK activation in regulating senescence (30, 56), we hypothesized that p90RSK activation could up-regulate T-bet expression. First, we investigated the relationship between the intensities of p90RSK S380 phosphorylation (p-p90RSK) and T-bet expression. p-p90RSK expression in B cells showed no significant difference with other cell types except NK cells (Figure 4A), and p-p90RSK expression did not increase after RT in the Ki67^–^DNMT3a^+^ naïve B cell subset (Figure 4B). But interestingly, we found a good correlation between p-p90RSK and T-bet in the samples from pre-RT, post-RT, and the combination of both (Figure 4C). Next, we compared T-bet expression with the intensities of total p90RSK (t-p90RSK). Although we could not find any significant correlation between T-bet and t-p90RSK in the samples from pre-RT, we found a significant correlation between T-bet and t-p90RSK in post-RT samples and a combination of both pre-and post-RT (Figure 4D). These data suggest that p90RSK activation and total p90RSK expression may play a role in T-bet expression.

**TABLE 1:** Profiling subsets, which showed a significant difference in indicated protein expression level between pre- and post-RT, using the Astrolabe Platform.

Cell subset	Channel name	MaxFc	*P*-value	adj. *P*-value
CD4– CD8– T Cell CD57lo Ki67lo CD38lo	c_Casp3	0.442926	1.82E-06	0.009163357
B Cell (CD27–) Ki67lo CD38lo DNMT3Ahi	Trx	0.78574	1.23E-05	0.015581951
CD4+ T Cell (Effector Memory) Ki67lo PD_1hi	HLA_DR	1.239167	6.47E-06	0.015581951
CD8+ T Cell (Central Memory) Ki67lo CD57lo PD_1hi	CD38	1.291662	1.24E-05	0.015581951
CD4+ T Cell (Effector Memory) Ki67lo PD_1hi	CD38	0.816107	2.07E-05	0.020827535
CD8+ T Cell (Effector Memory) Ki67lo CD57hi	CD38	0.906839	2.87E-05	0.024074599
CD4+ T Cell (Effector Memory) Ki67lo PD_1hi	Ki67	0.473691	4.13E-05	0.029698647
B Cell (Memory) Ki67lo T_betlo DNMT3Ahi	Ki67	0.694853	5.30E-05	0.033338777
CD4+ T Cell (Effector Memory) Ki67lo PD_1lo IL_7Ralo	HLA_DR	1.23317	8.55E-05	0.039773897
CD8+ T Cell (Central Memory) Ki67lo CD57lo PD_1hi	HLA_DR	1.105756	9.49E-05	0.039773897
CD8+ T Cell (EMRA) CD57hi PD_1lo T_bethi	CD38	0.843676	7.13E-05	0.039773897
CD8+ T Cell (EMRA) CD57hi PD_1lo T_betlo	CD38	0.967393	8.81E-05	0.039773897
B Cell (CD27–) Ki67lo CD38lo DNMT3Ahi	T_bet	1.168061	0.00013	0.042142932
B Cell (CD27–) Ki67lo CD38lo DNMT3Alo	c_Casp3	0.231743	0.000155	0.042142932
CD4+ T Cell (Effector Memory) Ki67lo PD_1lo IL_7Ralo	c_Casp3	0.367445	0.000159	0.042142932
CD4+ T Cell (Naive) CD25lo IL_7Ralo TET2lo	Trx	0.494799	0.000148	0.042142932
CD8+ T Cell (Central Memory) Ki67lo CD57lo PD_1hi	T_bet	0.530146	0.000155	0.042142932
CD8+ T Cell (Effector Memory) Ki67lo CD57lo PD_1lo	HLA_DR	0.999098	0.000129	0.042142932
CM–_unassigned CD57hi	CD38	0.845344	0.000121	0.042142932
CD4– CD8– T Cell CD57lo Ki67lo CD38lo	CD38	0.631695	0.000183	0.043743905
CD4+ T Cell (EMRA) IL_7Ralo PD_1lo CD25lo	c_Casp3	0.362187	0.000174	0.043743905

MaxFC, the maximum fold change out of all fold changes; adj. *P*. Val, A 2-sided false discovery rate (FDR) adjusted *P*-value.

**FIGURE 5:**
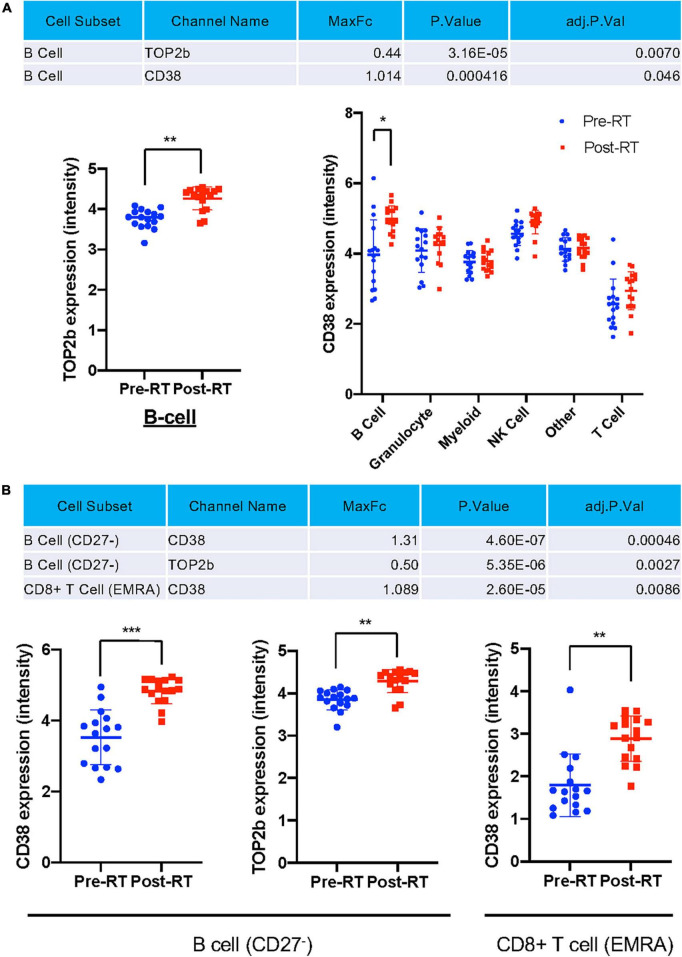
Only TOP2b and CD38 expression were significantly increased after RT at the cell subset level. **(A)** Only TOP2b and CD38 expression in B cells were increased after RT, and no significant change of studied molecules in cell types after RT compared to pre-RT level was observed. **(B)** CD38 and TOP2b expression in B cell (CD27^–^) and CD8^+^ T_EMRA_ subset pre- and post-RT. Mean ± SD, *adj. *P*-Val < 0.05, **adj. *P*-Val < 0.01, ***adj. *P*-Val < 0.001.

Astrolabe identified all protein expression levels examined in this study in each cell type (Figure 5A) and cell subtype (Figure 5B) and found that TOP2b and CD38 expression were increased after RT in the total B cells after RT (Figure 5A), and in the subsets of CD27^–^ B cells (Figure 5B). A significant increase in CD38 expression was also observed in CD8^+^ T_EMRA_ cells (Figure 5B). These data suggest a contribution of T-bet and CD38 expression to the induction of senescent phenotype after RT. Since TOP2b can protect against DNA damage, the increase of TOP2b expression after RT may contribute to DNA damage repair after RT.

### Radiation-induced T-bet^+^ memory and naïve B cells and myeloid cells by 90 kDa ribosomal S6 kinase activation

**FIGURE 6:**
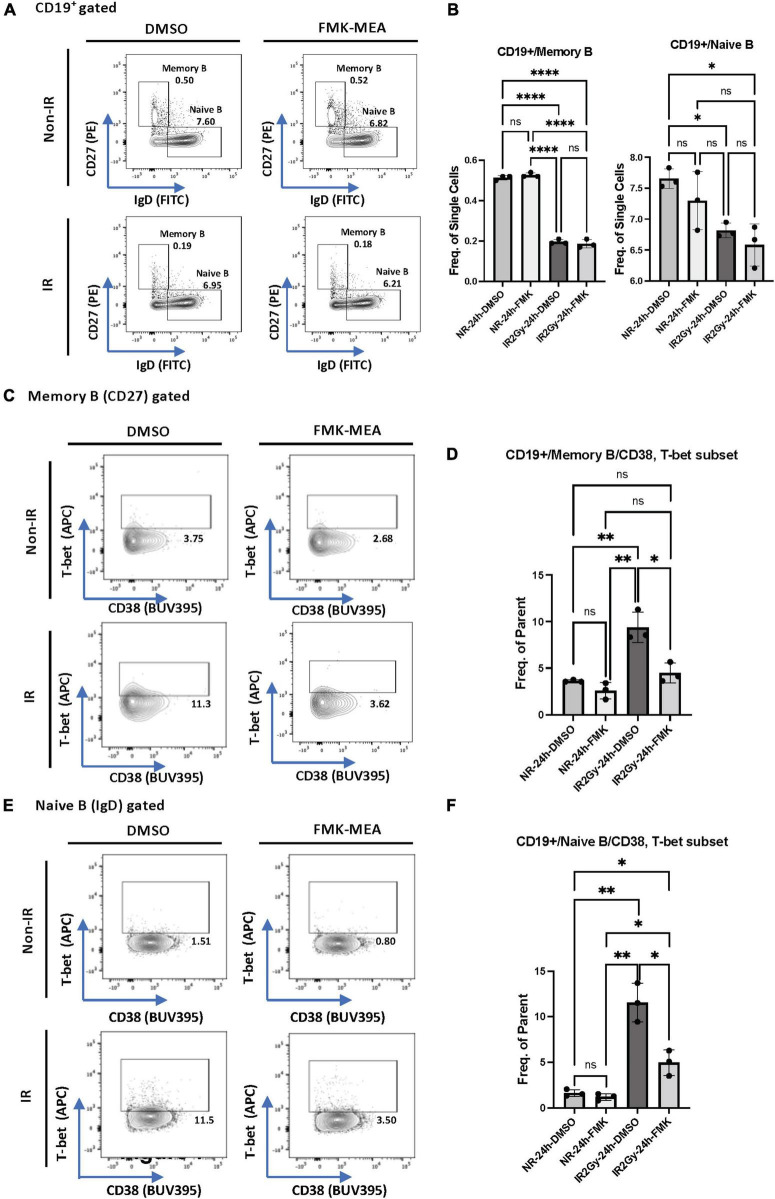
IR-induced CD38 + T-bet + CD19 + B cell *via* p90RSK activation. **(A)** Memory and naive B cell gating among CD19^+^ B cells. **(B)** Frequency of memory B cells **(Left)** and naive B cells **(Right)** among CD19^+^ cells 24 h after irradiation/non-irradiation with/without FMK-MEA. **(C)** Representative FACS plots for T-bet^+^ memory cells and their frequency among CD19^+^ B cells **(D)** 24 h after irradiation/non-irradiation with/without FMK. **(E)** Representative FACS plots for T-bet^+^ naive B-cells and their frequency among CD19^+^ B cells **(F)** 24 h after irradiation/non-irradiation with/without FMK. *N* = 3 for each group, and the experiments were done more than twice. Mean ± SD, *p < 0.05, ^**^p < 0.01, and ^****^p < 0.001.

**FIGURE 7:**
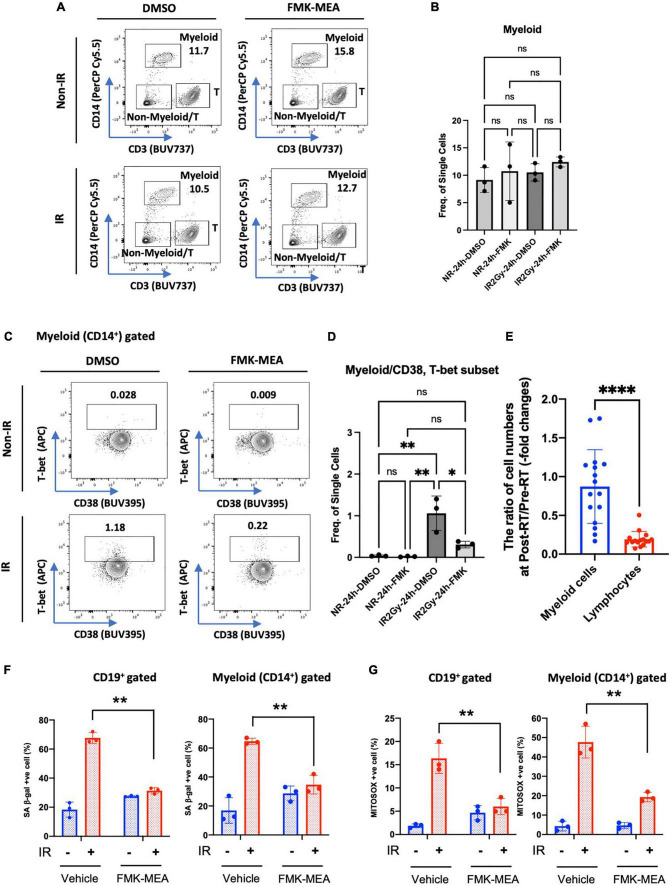
IR-induced CD38^+^/T-bet^+^ myeloid cells *via* p90RSK activation. **(A)** Representative FACS plots of myeloid T-cell distribution are depicted. **(B)** Myeloid cell frequency 24 h after irradiation/non-irradiation with/without FMK-MEA (10 μM). **(C,D)** Representative FACS plots **(C)** and their frequency **(D)** of T-bet expression in CD14^+^ myeloid cells in each condition. *N* = 3 for each group, and the experiments were done more than twice. Mean ± SD, *p < 0.05 and ^**^p < 0.01. **(E)** The ratio of cell numbers [myeloid cells **(Blue)** and lymphocytes **(Red)**] at pre-RT and post-RT. Mean ± SD, ^****^p < 0.001. **(F,G)** PBMCs were exposed to IR (2 Gy) with pre-treatment of FMK-MEA (10 μM) or vehicle for 1 hr. Senescent B cells and monocytes from PBMCs were measured using the C12FDG, a fluorogenic substrate for SA-β-galactosidase. Data shows the Quantification of positive C12FDG^+^ cells from CD19^+^ **(Left)** CD14^+^ **(Right)** cells. **(G)** Mitochondrial superoxide production was measured using the Mitosox dye in CD19^+^ (left) CD14^+^ (right) cells. Mean ± SD, *n* = 3, ^**^p < 0.01.

We next investigated whether p90RSK activation played a role in ionizing radiation (IR)-induced increase in CD38^+^T-bet^+^ cells. PBMCs from the healthy human subject were pre-treated with vehicle or FMK-MEA (a specific inhibitor of p90RSK), then exposed to IR (2 Gy). Previously, we have confirmed the specificity of FMK-MEA by using the Ambit/DiscoverX platform to screen 443 kinases (57). Twenty-four hours following the irradiation, the numbers and frequencies of naïve and memory B cells were decreased (Figures 6A,B). However, we found a significant increase of T-bet^+^ expression in memory and naïve B cells and this effect was inhibited by the p90RSK inhibitor FMK-MEA (Figures 6C–F). Since we have reported the role of SASP-induced CD38 expression in myeloid cells (32), we also investigated the impact of IR on CD38^+/^T-bet^+^ myeloid cells, and we found that pre-treatment of FMK-MEA also significantly inhibited IR-mediated CD38^+^/T-bet^+^ myeloid cell induction (Figure 7). These data suggest that IR increases CD38^+^/T-bet^+^ cells induction in both B-cells and myeloid cells even in the early phase after IR, dependent on p90RSK activation.

### Immunosenescence after radiation therapy and 90 kDa ribosomal S6 kinase activation

As shown in Supplementary Table 2 and Figure 7E, we found a significant decrease in lymphocytes number after RT. In contrast, myeloid cells numbers were relatively unaffected by RT, which resulted in relative myeloid skewing in the RT patients. To determine if RT induces immunosenescent in immune cells, we stained senescence-associated β-gal (SA-β-gal) staining and mitochondrial ROS (mtROS) production using healthy human PBMCs. Interestingly, CD19^+^ B cells and CD14^+^ myeloid cells in the PB showed significant increase of SA-β-gal^+^ cells and mtROS production after IR (Figures 7F,G). The increase of SA-β-gal^+^ cells and mtROS production was clearly blocked by pre-treatment of p90RSK inhibitor, FMK-MEA, in both B and myeloid cells. These results also suggest the crucial role of IR-induced p90RSK activation in immunosenescence.

## Discussion

In this study, we investigated the effects of RT on various senescence markers, DDR, efferocytosis, and clonal hematopoiesis drivers in immune cells. At 3 months after RT we observed a significant reduction in several immune cell subsets, particularly B cells.

Ki67^–^DNMT3a^+^ naïve B cells were the largest B cell subset. Although the frequency of this B cell (CD27^–^) subset was reduced after RT, we found a significant increase of T-bet expression that was correlated with phosphorylation of p90RSK in Ki67^–^DNMT3a^+^ naïve B cells. Furthermore, we also observed an increase in CD38 expression in B cells after RT. *In vitro* evidence suggested that p90RSK activation played a crucial role in the induction of CD38 and T-bet expression by IR in memory, naïve B, and myeloid cells. These data link RT-induced activation of p90RSK to upregulation of CD38^+^ and T-bet^+^ which are reported to have a role in immunosenescence (31, 32, 58).

The analysis using Astrolabe Platform of the CyTOF data revealed that the Ki67^–^DNMT3a^+^ naïve B cell subset was the largest subset of B cells, and had a unique response to RT. CD27 was used as a marker of memory B cells in humans based on the correlation of CD27 expression with somatic hypermutation in IgM^+^/IgD^+^ cells, and CD27 was constitutively expressed in approximately 40% of peripheral blood B cells in humans (59). CD27 is a member of the TNF-receptor family with the ligand of CD70, which is expressed on the surface of activated T cells. Activation of CD27 signaling plays a crucial role in maintaining long term immunological memory against T cell development antigen (60) by activating B cell expansion, differentiation, and antibody production (61–63). Therefore, the CD27^–^ B cell is recognized as a naïve B cell.

We examined various senescence and aging gene markers in various blood subsets using CyTOF, hoping to see the correlation between the irradiation and some changes of theses markers. The definition of the various subsets of T- and B- cells are listed in Supplementary Table 3. We focused B cells because only B cells showed significant change of some of these markers (CD38, TOP2β). In addition, the roles of DNMT3A in B cells have been reported. For example, DNMT3 regulates B cell lineage specification in the mouse model (64). Importantly, the depletion of DNMT3a in the B cell lineage results in the leukemic transformation of B1a cells in the CLL mouse model (65), and CD38 is a marker of human CLL B cell activation (66). Therefore, we assume that the B cell subset represented by lower expression of CD38 and higher expression of DNMT3a[i.e., (CD27^–^) Ki67^lo^ CD38^lo^ DNMT3a^hi^ cells] are a less proliferative B cell subset in accordance with low Ki67 expression. This was the dominant B cell subset before RT in thoracic cancer patients (Figure 3A). After RT, CD38 expression was increased regardless of Dnmt3a expression, suggesting that the residual B cells after RT were activated.

ABCs are antigen-experienced B cells that are characterized by a T-bet-mediated transcriptional program (67). ABCs plays a critical role in regulating immune response to infectious agents mediated by IgG2a/c and inflammatory cytokines (68, 69). However, the excessive accumulation of ABCs can trigger auto-immune reactions in response to self-antigen and may cause detrimental effects by inducing inordinate inflammation (70). In our current report, we found that T-bet expression was increased in the Ki67^–^DNMT3a^+^ naïve B cell subset, suggesting RT differentiated naïve B cells to ABC and induced inflammatory and senescence pathways. Also, the increase of T-bet expression in this largest but less active B cell subset (Ki67^–^DNMT3a^+^ naïve B cells) may significantly enhance the pre-mature aging process after RT by differentiating naïve B cells to ABC. Since systemic T-bet depletion inhibited atherosclerosis formation (38), it is possible that the increase of T-bet in B cells plays a role in elevating cardiovascular events after RT. Further investigation will be necessary.

It has been reported that CD38 expression in immune cells is increased in response to infection and inflammation, which subsequently induce inflammatory response (71). In rheumatoid arthritis (RA), the inhibition of CD38 activation by anti-CD38 monoclonal antibody has been effective to reduce RA symptoms and disease progression. In systemic lupus erythematosus (SLE) patients, the upregulation of CD38 expression in marginal zone-like IgD + CD27 + B cells has been reported (72), and the depletion of CD38 in pristane-induced lupus mouse model significantly improve the symptom (73). Thus, CD38 upregulation in B cells seems to be related to inflammatory status. In our current report, we found an increase in CD38 expression in B cells after RT in cancer patients. Since the essential role of CD38 in B cells in inflammatory responses has been reported (68, 69), these data suggest that NAD^+^ depletion induced by CD38 expression in B cells may initiate SASP. The induction of SASP in B cells may contribute to CVD in cancer patients after RT.

Lastly, we found a correlation between T-bet and p-p90RSK in the Ki67^–^DNMT3a^+^ naïve B cell subset. Furthermore, we found a crucial role of p90RSK activation in IR-induced CD38^+^/T-bet^+^ memory and naïve B cells and myeloid cells *in vitro*. Taken together, these data suggested that p90RSK activation induced by RT was involved in immunosenescence *via* upregulating CD38 and T-bet expression. Notably, the contribution of p90RSK activation and T-bet expression in immune cells to the process of atherosclerosis formation has been reported (38, 56), and to the best of our knowledge, this is the 1st report to show that p90RSK activation is required for T-bet induction. Interestingly, the potential role of T-bet on CD38 induction has been reported (74). Taken together, these data suggest that the activation of p90RSK induced by radiation plays a crucial role in T-bet-CD38 induction, resulting in immunosenescence and consequent atherosclerosis formation. Further investigation will be necessary.

## Data availability statement

The original contributions presented in this study are included in the article/Supplementary material, further inquiries can be directed to the corresponding author/s.

## Ethics statement

The Institutional Review Board of the University of Texas MD Anderson Cancer Center approved the clinical study protocol (#PA16-0971). The patients/participants provided their written informed consent to participate in this study.

## Author contributions

MI, HC, and SK performed experiments, analyzed data, and drafted manuscript. SK, AD, N-TL, EC, KK, VS, and L-LL supported the experiments and interpretation of the data. JH, XX, CR-G, S-CY, KS, SY, ZL, RN, E-aA., JB, NP, and JC contributed to the interpretation of the data and edited the manuscript. SL, MK, MY, and J-iA planned and generated the study design, obtained funding, interpreted data, and wrote the manuscript. All authors contributed to the article and approved the submitted version.

## Footnotes

1
https://www.nextprot.org

## Supplementary material

Supplementary Figure 1 The complete profiling subset heat map. Rows are cell profiling; columns are channels. Tile intensity is the median of the channel in each profiling.

Supplementary Figure 2 Profiling subsets of T cell, NKT cell, and monocyte revealed a significant difference in frequency between pre-RT and post-RT. **(A)** CD8^+^ T cell profiling subsets showed a significant difference between pre-RT and post-RT. **(B)** CD4^+^ T cell profiling subsets showed a significant difference between pre-RT and post-RT. **(C)** NKT cell profiling subset showed a significant difference between pre-RT and post-RT. **(D)** The monocyte profiling subset showed a significant difference between pre-RT and post-RT. Mean ± SD, **p* < 0.05 (or ^**^*p* < 0.01) by Student’s *t*-test, –log(FDR) > 0.5 and log(FC) < –1 or 1 < log(FC).

Supplementary Figure 3 T-bet expression in each cell type at pre-RT. Mean ± SD, ^***^*p* < 0.001. Statistical significance was assessed by Dunn’s multiple comparisons test.

Supplementary Table 1 CyTOF antibody panel and the markers with each protein function. To establish our CyTOF antibody panel, some cell surface markers were picked from the previous report (40).

Supplementary Table 2 Patient profiles. NSCLC, non-small cell lung cancer; SCLC, small cell lung cancer.

Supplementary Table 3 The definitions of each cell subset labeled by the Astrolabe platform.

Supplementary File 1 Expression level of each protein in each cell type at Pre-RT and Post-RT in samples obtained from 16 patients.

Supplementary File 2 Expression level of each protein in each cell subtype at Pre-RT and Post-RT in samples obtained from 16 patients.

Supplementary File 3 Expression level of each protein in each profiling subset at Pre-RT and Post-RT in samples obtained from 16 patients.

Supplementary File 4 Frequency of each profiling subset at Pre-RT and Post-RT in samples obtained from 16 patients.
