# Supplementary material for: Radiation therapy induces immunosenescence mediated by p90RSK
Source: Front Cardiovasc Med. 2022 Nov 7;9:988713. doi: 10.3389/fcvm.2022.988713 (PMC9680092; doi:10.3389/fcvm.2022.988713)
Supplement: Supplementary Figure 1 — The complete profiling subset heat map. Rows are cell profiling; columns are channels. Tile intensity is the median of the channel in each profiling. [file Data_Sheet_1.pdf]

Metal	Marker			Surface or Internal
89Y	CD45	DVS-Fluidigm	3089003B	S
111Cd	p16	Abcam	ab54210 (100 uL)	I
112Cd	Trx	Proteintech	14999-1-AP (150 uL)	I
114Cd	p-jak2	Abcam	ab219728	I
115In	CD57	BioLegend	322325	S
116Cd	TET2	Invitrogen	MA5-38640	I
141Pr	phospho-P90RSK(S380)	ABclonal	AP0562	I
142Nd	Caspase 3, cleaved	DVS-Fluidigm	3142004A	I
146Nd	CD8a	BioLegend	301002	S
147Sm	TOP2b	R&D	MAB6348 (100 ug)	I
148Nd	CD27	BioLegend	124202	S
149Sm	p53	BD	554294	I
150Nd	CD11c	BioLegend	337221	S
151Eu	CD123	BD	554527	S
152Sm	jak2	US Biological	37177	I
153Eu	CD4	BioLegend	357402	S
154Sm	T-bet	BioLegend	644825	S
155Gd	CD25	BD	555430	S
156Gd	TIM-3	BioLegend	345002	S
158Gd	CD33	DVS-Fluidigm	3158001B	S
159Tb	P90RSK	StressMarq	SPC-147F	I
160Gd	CD14	BioLegend	301802	S
162Dy	CD56	BD	559043	S
163Dy	CD45RA	BioLegend	304102	S
164Dy	Tyro3	Novus	NBP1-28635 (0.1 mL)	I
165Ho	CD19	DVS-Fluidigm	3165025B	S
166Er	CD41	BioLegend	303702	S
167Er	CD38	BioLegend	303502	S
168Er	Ki67	BD	556003	I
169Tm	CD279, PD-1	Miltenyi	130-096-168	S
170Er	DNMT3A	Novus	NB120-13888 (0.1mg)	I
171Yb	CD68	DVS-Fluidigm	3171011B	I
173Yb	CD11b	BioLegend	301302	S
174Yb	HLA-DR 174Yb (MDA)	BioLegend	307602	S
175Lu	CD3	BioLegend	300443	S
176Yb	CD127, IL-7Ra	DVS-Fluidigm	3176004B	S
209Bi	CD16	DVS-Fluidigm	3209002B	S
191Ir	Ir DNA-Intercalator	DVS-Fluidigm	201192A	nucleus
193Ir	Ir DNA-Intercalator	DVS-Fluidigm	201192A	nucleus
103Rh	Rh DNA-intercalator	DVS-Fluidigm	201103A	Viable cells

Table S1

	n	Mean (SD)
Sex		
Male	16	100%
Female	0	0%
Diagnosis		
Esophagus cancer	14	87.50%
SCLC	2	14.30%
Age	16	65.00 (8.48)
RT modality		
Proton	3	18.8%
VMAT	11	68.8%
3-D	1	6.3%
IMRT	1	6.3%
RT total dose	16	53.20 (6.30)
RT fraction dose	16	1.88 (0.20)
RT fraction	16	28.25 (0.68)
Neutrophils number (K/μl)		
Pre-RT	16	5.13 (1.92)
End-RT	16	3.78 (1.73)*
Lymphocytes number (K/μl)		
Pre-RT	16	1.76 (0.48)
End-RT	16	0.32 (0.13)**
Monocytes (K/μl)		
Pre-RT	16	0.72 (0.20)
End-RT	16	0.67 (0.32)
Eosinophils (K/μl)		
Pre-RT	16	0.19 (0.11)
End-RT	16	0.12 (0.13)
Basophils (K/μl)		
Pre-RT	16	0.05 (0.02)
End-RT	16	0.03 (0.02)
Risk factor of CVD		
Hypertension	9	56.3%
Hyperlipidemia	10	62.5%
Diabetes	5	31.3%
Obesity (BMI>30kg/m2)	5	31.3%
Past history of CVD	6	37.5%

Table S2

Compartment	Cell subset	Description
T cell	NKT cell	CD3+CD19-CD33-CD41-CD56+
T cell	CD8+T cell (Naïve)	CD3+CD19-CD33-CD41-CD56-, CD4-CD8a+, CD45RA+, CD27+
T cell	CD8+ T cell (EMRA)	CD3+CD19-CD33-CD41-CD56-, CD4-CD8a+, CD45RA+, CD27-
T cell	CD8+ T cell (Efector Memory)	CD3+CD19-CD33-CD41-CD56-, CD4-CD8a+, CD45RA-, CD27-
T cell	CD8+ T cell (Central Memory)	CD3+CD19-CD33-CD41-CD56-, CD4-CD8a+, CD45RA-, CD27+
T cell	CD4+T cell (Naïve)	CD3+CD19-CD33-CD41-CD56-, CD4+CD8a-, CD45RA+, CD27+
T cell	CD4+T cell (EMRA)	CD3+CD19-CD33-CD41-CD56-, CD4+CD8a-, CD45RA+, CD27-
T cell	CD4+T cell (Effector Memory)	CD3+CD19-CD33-CD41-CD56-, CD4+CD8a-, CD45RA-, CD27-
T cell	CD4+T cell (Central Memory)	CD3+CD19-CD33-CD41-CD56-, CD4+CD8a-, CD45RA-, CD27+
T cell	CD4+CD8+ T cell	CD3+CD19-CD33-CD41-CD56-, CD4+CD8a+
T cell	CD4-CD8- T cell	CD3+CD19-CD33-CD41-CD56-, CD4-CD8a-
Other	CM- HLADR+	CD3-CD19-CD33-CD41-CD56-, CD123- HLA DR+
Other	CD41+	CD3-CD19-CD33-CD41+CD56-
NK cell	NK Cell (CD56+ CD16+)	CD3-CD19-CD33-CD41-CD56+, CD16+
NK cell	NK Cell (CD56+ CD16-)	CD3-CD19-CD33-CD41-CD56+, CD16-
Myeloid	Monocyte (CD14+ CD16+)	CD3-CD19-CD33+CD41-, CD14+, CD16+
Myeloid	Monocyte (CD14+ CD16-)	CD3-CD19-CD33+CD41-, CD14+, CD16-
Myeloid	Monocyte (CD14- CD16+)	CD3-CD19-CD33+CD41-, CD14-, CD16+
Myeloid	Dendritic Cell (Plasmacytold)	CD3-CD19-CD33-CD41-CD56-, CD11c-CD123+HLA DR+
Myeloid	Dendritic Cell (Conventional)	CD3-CD19-CD33+CD41-, CD14-, CD16-
Granulocyte	Granulocyte (Basophil)	CD3-CD19-CD33-CD41-CD56-, CD123+HLA DR-
B cell	B cell (Plasmablast)	CD3-CD19+CD33-CD41-CD56-, CD27+ CD38+
B cell	B cell (Memory)	CD3-CD19+CD33-CD41-CD56-, CD27+, CD38-
B cell	B cell (CD27-)	CD3-CD19+CD33-CD41-CD56-, CD27-

Table S3

Figure S1

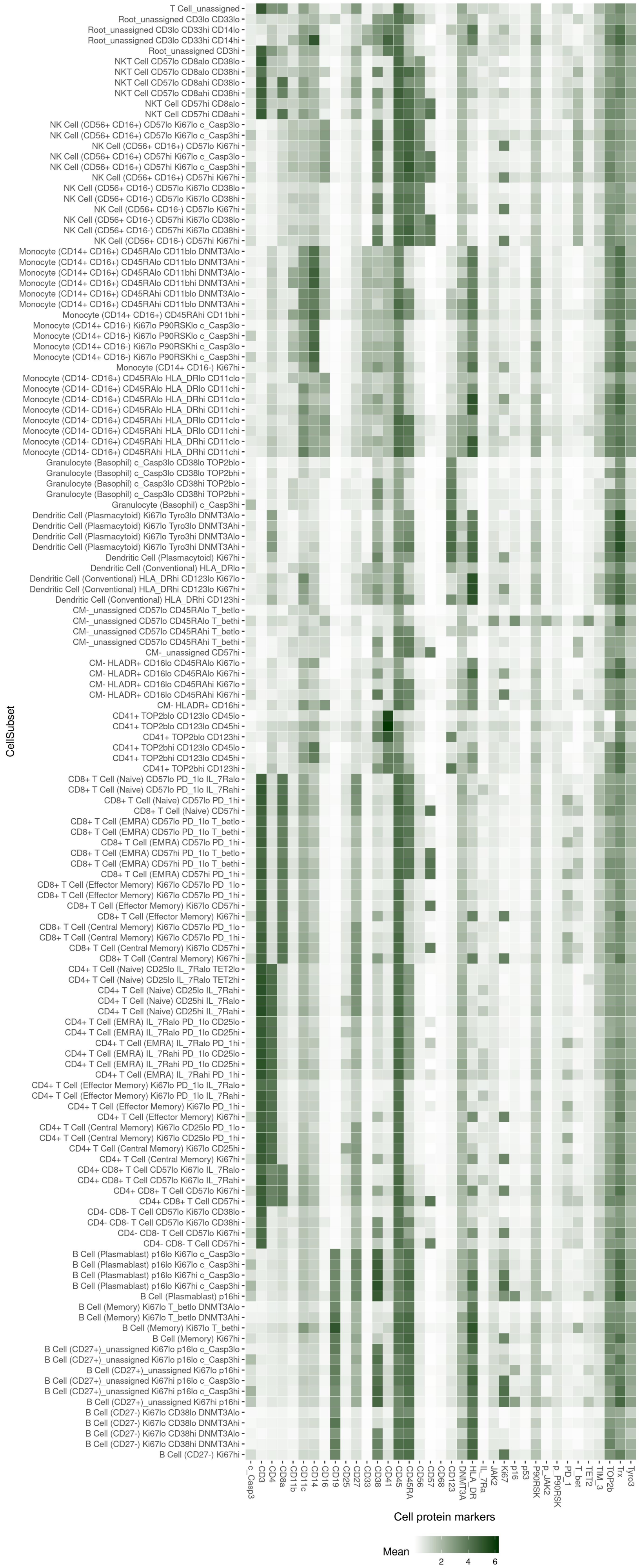
CellSubset
Cell protein markers
Mean
2
4
6
c_Casp3
CD3
CD4
CD8a
CD11b
CD11c
CD14
CD16
CD19
CD25
CD27
CD33
CD38
CD41
CD45
CD45RA
CD56
CD57
CD68
CD123
DNMT3A
HLA_DR
IL_7Ra
JAK2
Ki67
p16
p53
P90RSK
p_JAK2
p_P90RSK
PD_1
T_bet
TET2
TIM_3
TOP2b
Trx
Tyro3

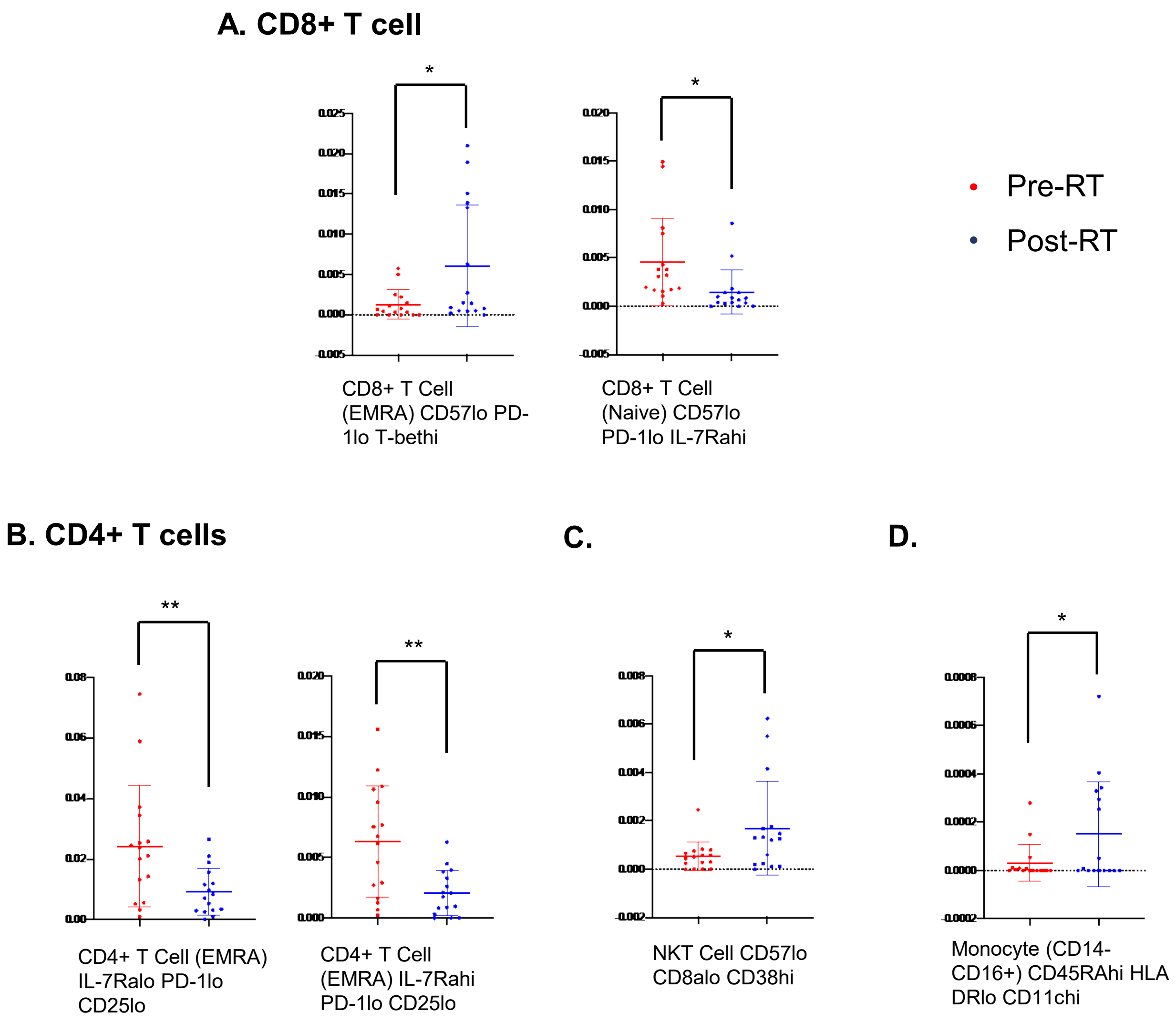

A. CD8+ T cell
*
0.025
0.020
0.015
0.010
0.005
0.000
-0.005
CD8+ T Cell (EMRA) CD57lo PD-1lo T-bethi
*
0.020
0.015
0.010
0.005
0.000
-0.005
CD8+ T Cell (Naive) CD57lo PD-1lo IL-7Rahi
Pre-RT
Post-RT
B. CD4+ T cells
**
0.08
0.06
0.04
0.02
0.00
CD4+ T Cell (EMRA) IL-7Ralo PD-1lo CD25lo
**
0.020
0.015
0.010
0.005
0.000
CD4+ T Cell (EMRA) IL-7Rahi PD-1lo CD25lo
C.
*
0.008
0.006
0.004
0.002
0.000
-0.002
NKT Cell CD57lo CD8alo CD38hi
D.
*
0.0008
0.0006
0.0004
0.0002
0.0000
-0.0002
Monocyte (CD14- CD16+) CD45RAhi HLA DRlo CD11chi

Figure S2

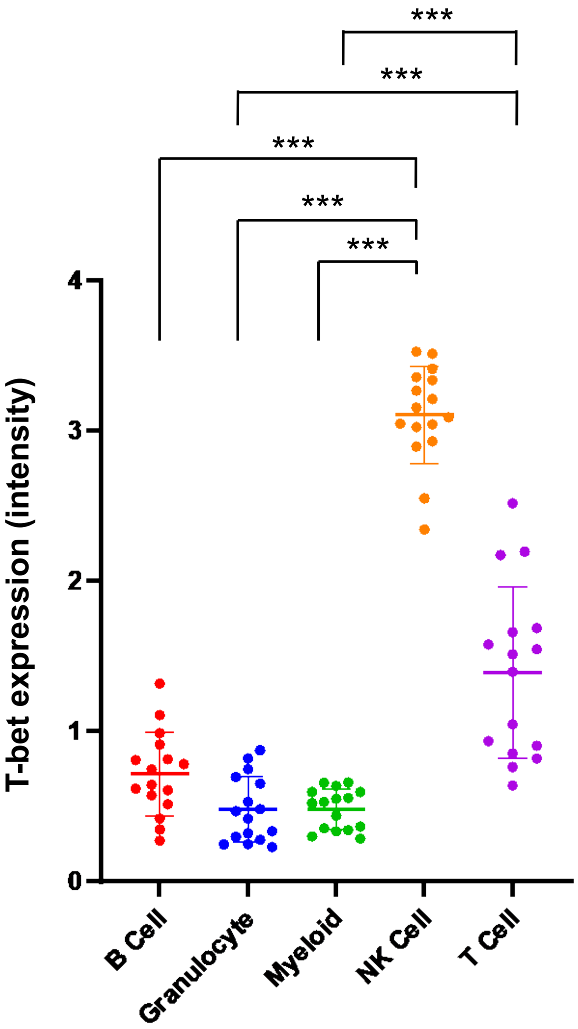
***
***
***
***
***
4
3
2
1
0
T-bet expression (intensity)
B Cell
Granulocyte
Myeloid
NK Cell
T Cell

Figure S3
